# SOCS molecules: the growing players in macrophage polarization and function

**DOI:** 10.18632/oncotarget.19940

**Published:** 2017-08-04

**Authors:** Dexi Zhou, Lu Chen, Kui Yang, Hui Jiang, Wenke Xu, Jiajie Luan

**Affiliations:** ^1^ Department of Pharmacy, Yijishan Hospital, Wannan Medical College, Wuhu, Anhui Province, China; ^2^ Laboratory of Clinical Pharmacy, Wannan Medical College, Wuhu, Anhui Province, China

**Keywords:** macrophage polarization, cytokines signaling, suppressors of cytokine signaling

## Abstract

The concept of macrophage polarization is defined in terms of macrophage phenotypic heterogeneity and functional diversity. Cytokines signals are thought to be required for the polarization of macrophage populations toward different phenotypes at different stages in development, homeostasis and disease. The suppressors of cytokine signaling family of proteins contribute to the magnitude and duration of cytokines signaling, which ultimately control the subtle adjustment of the balance between divergent macrophage phenotypes. This review highlights the specific roles and mechanisms of various cytokines family and their negative regulators link to the macrophage polarization programs. Eventually, breakthrough in the identification of these molecules will provide the novel therapeutic approaches for a host of diseases by targeting macrophage phenotypic shift.

## INTRODUCTION

Cytokines profiles are now well-defined in the pathophysiology of multiple diseases mainly by influencing the growth, proliferation, differentiation of a variety of cell types [1]. Theses cytokines signaling pathways are triggered as a consequence of enhanced interaction of cytokines with their specific cell surface receptors, which leads to the activation of intracellular molecules, such as Janus kinases (JAKs) and signal transducers and activators of transcription (STATs) [2, 3]. Studies have reported that macrophages are important immune cells and function as the direct target of some cytokines, which might contribute to the pathogenesis of many diseases [4, 5]. Monocyte-macrophage lineage has long been recognized as heterogeneous cell type that could undergo a continuum from M1 to M2 activation states depending on the microenvironment signals [6, 7]. The M1/M2 subpopulation represents the dynamic shift between inflammatory and reparative macrophages, which significantly contribute to either beneficial or detrimental effects in different diseases [8, 9]. As expected, these distinct and opposing subsets are often exposed to the various cytokines stimuli, for example interleukins (ILs), interferons (IFNs) and growth factors [10–12].

In response to pathogens or injury, cytokines and intracellular signaling pathways may promote the M1/M2 macrophage polarization in cancer and other disorders [13–15]. Suppressors of cytokine signaling (SOCS) proteins act as inducible negative feedback regulators of cytokine signaling by a generic mechanism of targeting associated proteins for degradation [16, 17]. Profound new discoveries have showed that the disparate SOCS family molecules may serve as the molecular switch that controls immune activation/suppression and M1/M2 macrophage polarization [18, 19]. Herein, this review provides an overview of the emerging roles and mechanisms of SOCS proteins in cytokines-induced M1/M2 macrophage polarization. Taken together, an updated understanding of the fate of SOCS-directed macrophage polarization and function could guide the development of novel therapeutic targets for various diseases.

## THE CLASSIFICATION, STRUCTURE, AND ACTION MECHANISM OF SOCS FAMILY OF PROTEINS

At present, the mammalian SOCS family of proteins is believed to consist of eight members: SOCS1-7 and the alternatively named cytokine-inducible Src homology 2 (SH2)-containing protein (CIS) [20, 21]. The SOCS/CIS family is a group of intracellular proteins, several of which downregulate cytokines signaling following cytokines engagement of their specific receptors complex through a negative feedback loop [22].

**Figure 1:**
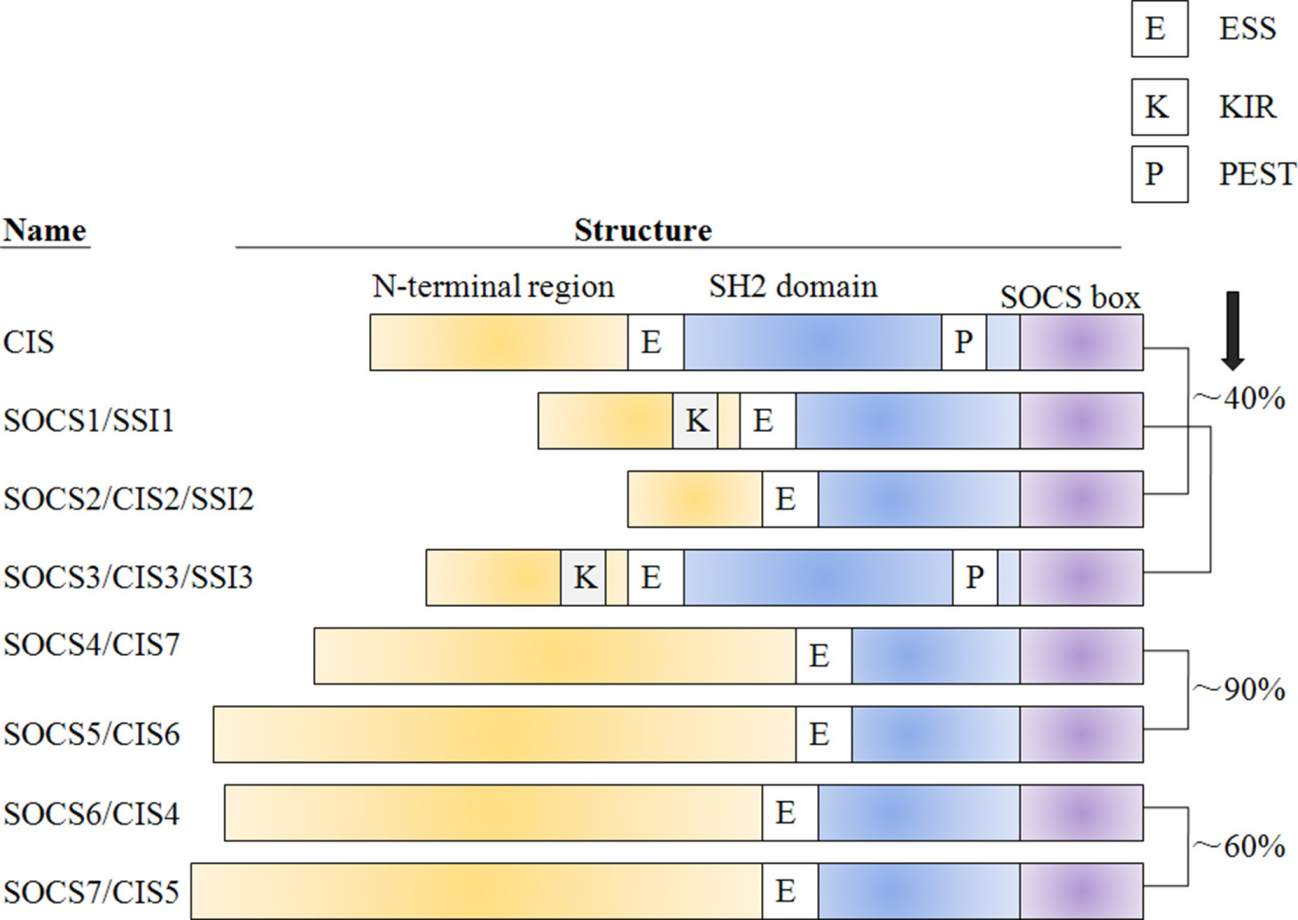
The classification and domain structure of the SOCS protein family All of the eight SOCS-family members have a central SH2 domain,an amino-terminal domain of variable length and a 40-amino-acid motif at the carboxy terminus that is known as the SOCS box. Although there is sequence homology between all family members-particularly in the SOCS box and SH2 domain-CIS and SOCS2, SOCS1 and SOCS3, SOCS4 and SOCS5, and SOCS6 and SOCS7 have marked pair-wise homology across the entire protein sequence, as indicated. In SOCS1 and SOCS3, a kinase-inhibitory region (K) adjacent to the SH2 domain that is required for high-affinity binding to Jaks and the inhibition of kinase activity has also been defined. The alternative nomenclature for each SOCS protein is given in parentheses. CIS, cytokine-induced SH2 protein; JAB, Janus kinase(Jak)-binding protein; NAP4, Nck, Ash and phospholipase-C binding protein; SH2, SRC-homology 2; SOCS, suppressor of cytokine signalling; SSI, Stat-induced Stat inhibitor; Stat, signal transducer and activator of transcription.

Lines of evidence suggest that SOCS/CIS family negatively regulates the cytokines signaling is largely attributing to their domain structure characteristics [23]. Each SOCS protein shares a common modular organization that generally contains three differentially conserved domains: an amino (N)-terminal region of varied length and amino acid sequence (low conserved), a central SH2-domain (conserved) and a carboxy (C)-terminal 40 amino acid module, e.g. SOCS box motif (highly conserved), which has been well-described in previously published literatures [24, 25]. Particularly, the SOCS3 and CIS-SH2 domains exist a 35-residue (PEST) (proline-, glutamicacid-, serine- and threonine-rich)-motif that inserted between the α β helix and the BG loop, which can affect the SOCS3 turnover and stability [26]. Another feature of the SOCS family is the extended SH2 sequence called (ESS) that is important for the interaction with phosphotyrosine residues on the target protein [27]. There exists an amino acid sequence homology in pairs between all SOCS family members: CIS and SOCS2, SOCS1 and SOCS3, SOCS4 and SOCS5, and SOCS6 and SOCS7, which have marked pair-wise homology across the entire protein sequence [28, 29]. However, several important distinctions among different SOCS members are also unambiguous in view of firstly based on a short(CIS, SOCS1-3) or long, N-terminal region(SOCS4-7) and secondly, it is the former group, which are most clearly induced in response to cytokine signaling and act in a classical negative-feedback loop [30] (Figure 1).

**Figure 2:**
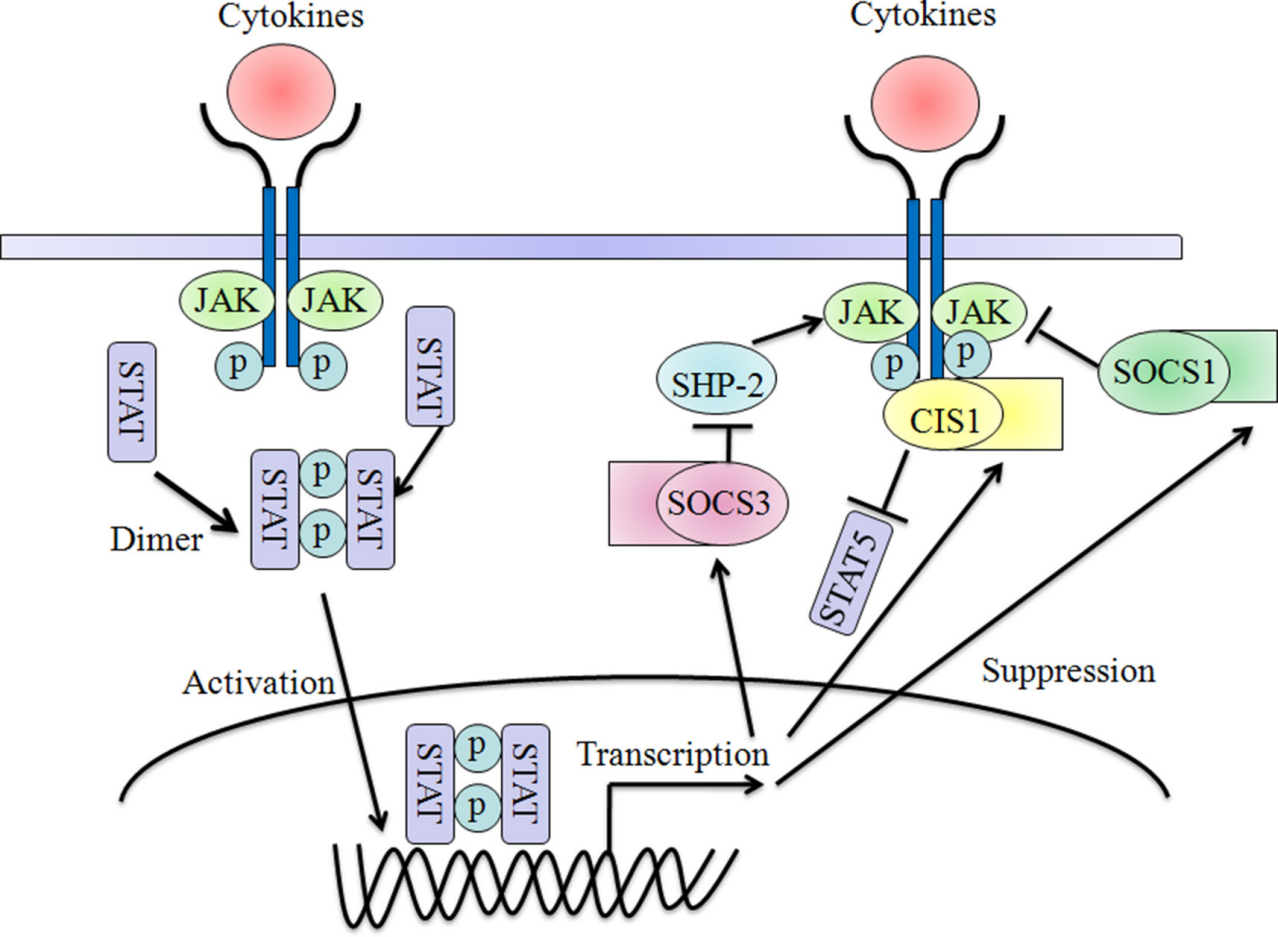
Negative-feedback loop regulation of cytokines signaling by SOCS proteins Cytokine stimulation activates the Jak-Stat pathway, leading to the induction of CIS, SOCS1 and SOCS3. CIS, SOCS1 and SOCS3 could inhibit signaling by different mechanisms: SOCS1 binds to the JAKs and inhibits catalytic activity; SOCS3 binds to JAK-proximal sites on cytokine receptors and inhibits JAK activity; and CIS blocks the binding of Stats to cytokine receptors. Both SOCS1 and SOCS3 contain a kinase inhibitory region (KIR) for the suppression of Jak tyrosine kinase activity.

There are mainly two kinds of action modes that are necessary for the proper regulatory functions of CIS-SOCS-family proteins. The first approach of all SOCS proteins to degrade the targets is dependent on the ubiquitin pathway [31]. The SOCS box interacts with elongin B and C, cullin-5 and RING-box-2 (RBX2) to recruit E2 ubiquitin transferase and E3 ubiquitin ligases complex. This could lead to the polyubiquitylation of bound signaling proteins, and their consequent proteasomal degradation, resulting in the termination of signaling [32, 33]. Second, both SOCS1 and SOCS3 can inhibit the catalytic-activity function of JAK tyrosine kinase by acting as a pseudosubstrate and competing with substrates through their SH2 domain and kinase inhibitory region (KIR), which locates N-terminal domain [34, 35] (Figure 1). Meanwhile, CIS and SOCS2 have been thought to act by competing with STAT proteins for binding to phosphorylated tyrosine residues within the receptor cytoplasmic domains [36]. In contrast, the functions and mechanisms of SOCS4-7 will be discussed later in this review (Figure 2).

## CYTOKINES SIGNALING AND MACROPHAGE POLARIZATION

Within differential cytokines microenvironment, macrophage could undergo polarization toward M1 or M2 phenotype. Though the mechanisms of cytokines signaling controlling macrophage polarization remain not fully clear, we discuss some recently important findings regarding the roles of cytokines signaling in the regulation of macrophage polarization.

## INTERLEUKINS (ILS)

Interleukins (ILs) are a wide range of peptidic substances released by a variety of cell types in response to various inflammatory processes [37]. They are known as soluble factors and thought to have a critical role in the modulation of many immune pathways [38, 39]. Remarkably, it has now been approved that monocytes and macrophages M1/M2 phenotype formation required after the ILs stimulation.

It has been widely accepted that IL-4 or/and IL-13 can stimulate the macrophage into the M2 subpopulation [10, 40]. IL-4Rα binding of IL-4 activates JAK1 and JAK3, which lead to the activation and translocation of STAT6 into nucleus where it binds to the promoter region of target M2 genes [41, 42]. IL-4-provoked M2 functions are largely mediated by the activation of IL-4/STAT6 signaling pathway and enhanced Arg-1 production [43, 44]. Furthermore, IL-13 receptor alpha 1 (IL-13Rα1) specifically heterodimerizes with the IL-4Rα chain to form a type II heteroreceptor, which is able to phosphorylate STAT6 efficiently and controls the differentiation and function of M2 macrophages [45, 46]. Thus, utilizing an IL-4/STAT6-dependent mechanism to shift macrophage polarization to the M2/anti-inflammatory phenotype might ameliorate inflammatory and autoimmune diseases in clinical treatment. IL-5 activity inhibition may lead to the substantial reduction of IL-13-triggered M2 responses, which were associated with increased production of the cytokine IFN-γ [47]. Conversely, tumor associated macrophages (TAMs) from the STAT6-null mice showed a functional M1 phenotype upon activation as characterized by i.e. NO production, which firmly indicated that JAK1, 3/STAT6 signaling pathway are essential for the M2 activation [48, 49]. IL-4 in combination with IL-13 has been widely used as the inducers *in vitro* of skewing monocytes/macrophages into the M2 polarization. However, IL-4 and IL-13 signaling in monocytes/macrophages might differentially regulate the expression of several inflammatory genes starting from IL-4/IL-13 cytokine receptors to ultimately control Jak/Stat-mediated signaling pathways [50]. IL-13 utilizes both IL-4Rα/Jak2/Stat3 and IL-13Rα1/Tyk2/Stat1/Stat6 signaling pathways, while IL-4 can use only the IL-4Rα/Jak1/Stat3/Stat6 cascade to promotethe expression of some critical inflammatory genes [51]. These conclusions provide novel insights into the mechanisms and functions of alternatively activated monocytes/macrophages stimulated by IL-4 and IL-13, which have important implications for the potential treatment of multiple inflammatory diseases.

**Figure 3:**
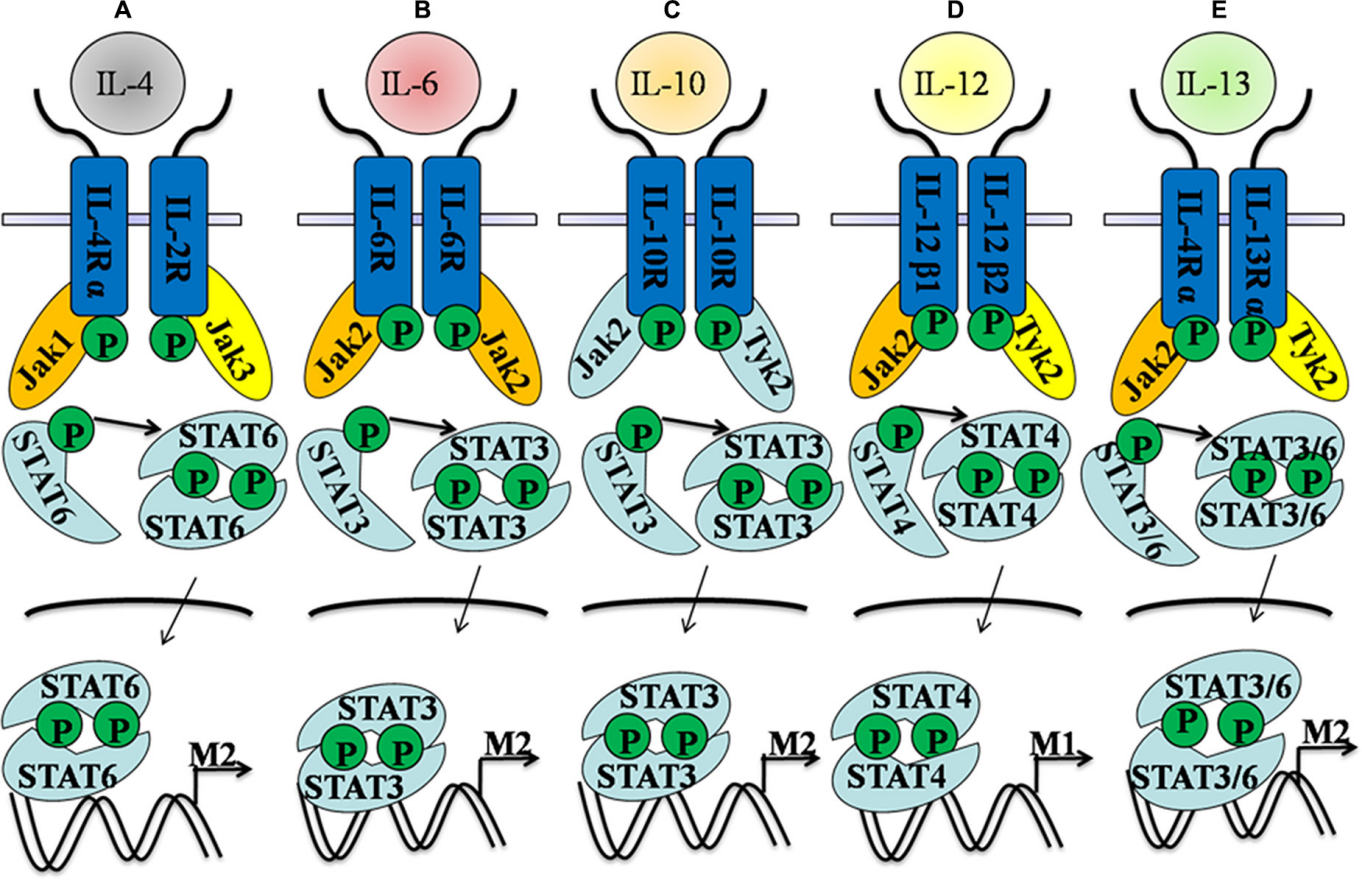
Different cytokines stimuli direct the effects of M1- and M2-like polarization Here, we summarize key signaling mediators and receptors in common and distinct pathways. (**A**) IL-4-stimulated Stat6 activation is mediated by Jak1, which is required for M2-like polarization. (**B**) IL-6-treated macrophage polarization is induced by the activation of Jak2/Stat3 signaling pathway. (**C**) Jak2 and Tyk2 are required for Stat3 activation in IL-10-dependent M2-like polarization. (**D**) Jak2 controls Stat4 activation in IL-12-treated monocytes, which could induce M1-like polarization. (**E**) Jak2 and Tyk2 are the upstream regulators of Stat3 and Stat6 activation in IL-13-stimulated monocytes, which eventually induce the M2-like polarization.

In addition, though IL-6 is commonly recognized as a pro-inflammatory mediator and is associated with the pathogenesis of many inflammatory processes, the pleiotrophic nature of IL-6 are also controversial [52, 53]. IL-6 is considered to assign an unexpected homeostatic role to limit inflammation and as an important inducer in promoting M2 polarization, which is mainly dependent on the upregulation of the IL-4Rα [54]. IL-6 could induce the expression of the receptor for IL-4 and augment the response to IL-4 in macrophages, but IL-6ra^Δmyel^ mice are resistant to IL-4-mediated M2 polarization and exhibit enhanced susceptibility to lipopolysaccharide (LPS)-induced endotoxemia [55]. Besides, the anti-inflammatory cytokine IL-10 promotes M2 polarization through the induction of p50 NF-κB homodimer, c-Maf, and STAT3 activities [56]. On the contrary, the M1 subtype is a phenotype characterized by the marked increase in cytokines(IL-1β, IL-12) and decrease in cytokines(IL-4, 10 and 13) [57, 58]. IL-12p40 and L-12p35 bind to IL-12Rβ1 and β2, respectively, which results in transphosphorylation of associated JAKs (JAK2 and TYK2) and then leads the activation and translocation of STAT4 into nucleus where they bind to STAT binding sites in the IFN-γ promoter of targeting M1 genes [59]. In conclusion, different STATs seem to be pivotal factors and relevant to the in M1/ M2 balance (Figure 3).

## OTHER CYTOKINES

Chemokines are a superfamily of small proteins with a crucial role in the polarized immune responses [60]. Particularly, chemokines and their receptors are able to trigger the differentiation and activation of mononuclear phagocytes by influencing the expression of functionally relevant and polarization-associated genes [61]. The crosstalk between chemokines and other cytokines has been reported to play critical roles in macrophage polarization. For example, CCL2 and IL-6 were found to contribute to the survival of CD11b^+^ myeloid monocytes recruited to the tumor microenvironment and skew the phenotype toward tumor-promoting CD14^+^/CD206^+^ M2-type macrophages [62]. CCL2-CCR2 axis was found to down-regulate the extent of pro-inflammatory M1 polarization by influencing the polarization-associated genes, including TNF-α, IL-6, granulocyte macrophage colony-stimulating factor (GM-CSF) and macrophage colony-stimulating factor (M-CSF) [63, 64]. GM-CSF and M-CSF are commonly recognized as critical factors controlling the M1 and M2 polarization, respectively [65]. GM-CSF favors the M1 phenotype through the activation of Jak2-STAT5 signalling [66], but M-CSF-dependent M2 phenotype is largely skewed by the activation of STAT3 [67]. Moreover, activin A preferentially released by M1 (GM-CSF) macrophages, and activin A-initiated Smad2 and prolyl hydroxylase PHD3 signaling both skew macrophage polarization toward the acquisition of a proinflammatory phenotype [68, 69]. Investigation of GM-CSF knockout alveolar macrophages (AMs) revealed intrinsic overexpression of IFN-γ, a potent inducer of the M1 phenotype, which as a causative factor for activin A, iNOS, CCL5, and IL-6 upregulation [70]. Examination of M2 markers in GM-CSF knockout mice are also simultaneously elevated, suggesting a unique mix of M1-M2 macrophage phenotypes in GM-CSF knockout mice. Besides, TGF-β is believed to control the M2-like polarization in part through the activation of Jak-STAT signaling [71]. Apparently, the activation of various cytokines signaling has been shown to act as the critical modulator of macrophage polarization based on the aforementioned researches. Thus, the molecules that could switch M1/M2 polarization derived from the cytokines signaling might provide a basis for macrophage-centered therapeutic strategies.

## SOCS PROTEINS FAMILY AND MACROPHAGE POLARIZATION

### SOCS1

SOCS1 is one of the best-studied SOCS proteins and is initially reported as a molecule induced by STATs [34, 72]. The expression of SOCS1 has now been confirmed to be induced by diverse cytokines, including insulin and LPS [73]. It is a well-known negative regulator of JAKs-STATs by accessing the activation loop of JAKs with its KIR domain and these effects are associated with the attenuated signaling of LPS, IFN-γ and IL-4 in many diseases [74–76].

**Figure 4:**
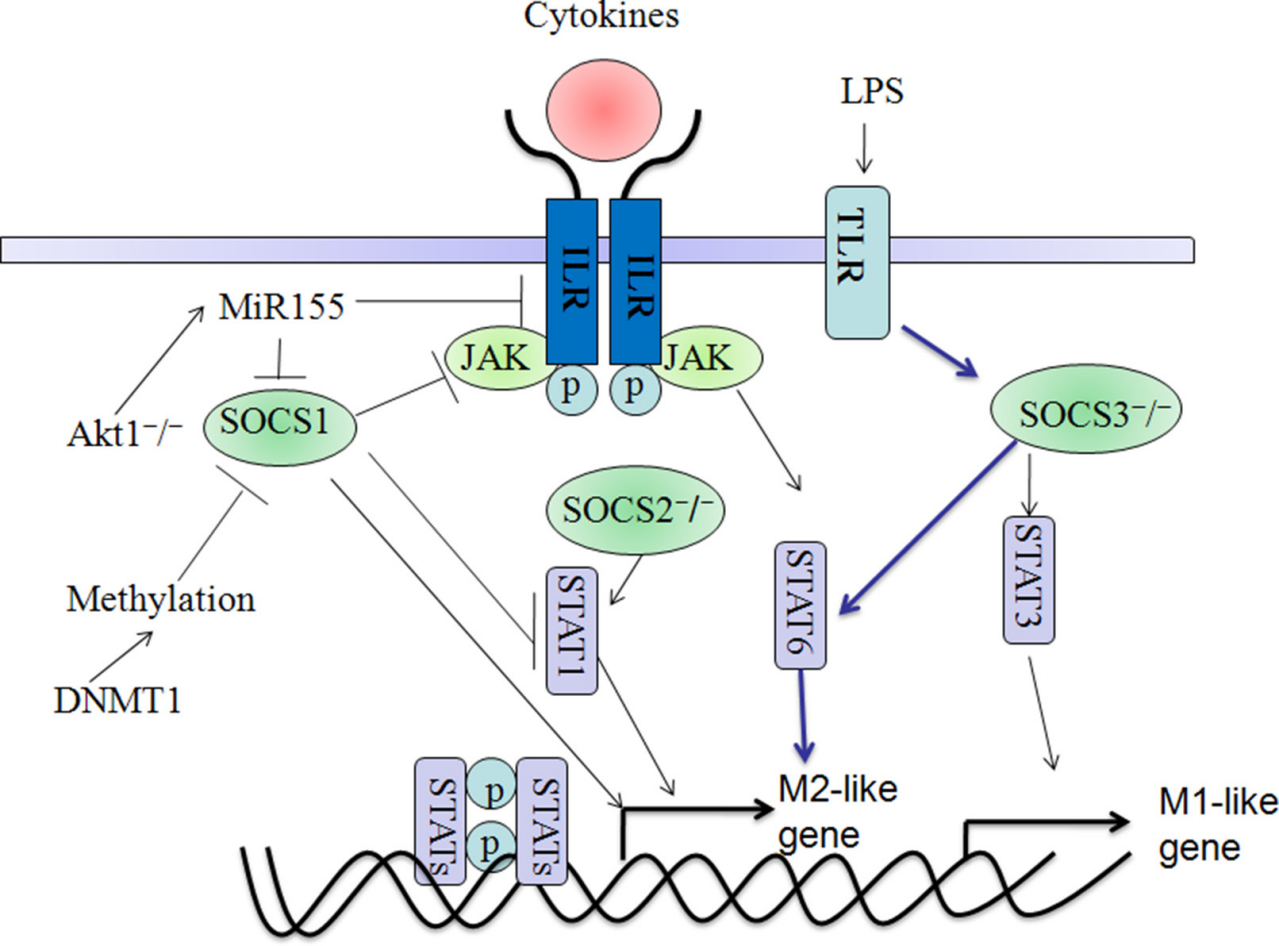
Functions and molecular mechanisms of SOCS1, SOCS2 and SOCS3 are implicated in directing macrophage polarization Firstly, SOCS1 suppresses Stat1 and stimulates the M2-type polarization which could be influenced by different molecular mechanisms. Particularly, Akt1 ablation might influence MiR-155 expression and promote the M1-like polarization by targeting and inhibiting SOCS1. Moreover, DNMT1-mediated the methylation of SOCS1 could influence the M2-like macrophage. Secondly, SOCS2 knock out might stimulate M2-like polarization via the activation of Stat1. Thirdly, SOCS3 knock out could stimulate the M2- and M1-like polarization via the activation of Stat6 and Stat3, respectively. However, there seems to be still elusive about the SOCS proteins in the regulation of macrophage polarization and the real situation is much more complicated.

SOCS1 has been reported to exert a significant role in the regulation of macrophage polarization and function [77, 78]. Macrophage phenotypes alterations have been shown to be influenced by the epigenetic mechanism by which SOCS1 plays as a capacitor for M1/M2 polarization. Upregulation of DNA methyltransferases1 (DNMT1) appears to be linked with the SOCS1hypermethylation, which lead to the activation of JAK2/STAT3 pathway and the releasement of LPS-induced pro-inflammatory cytokines(TNF-α, IL-6) in macrophages [79, 80]. This result indicated that loss of SOCS1 expression might direct the pro-inflammatory M1 effect of macrophage by activating the JAK/STAT pathway. In addition, microRNAs (miRs) were also shown to play pivotal roles in the SOCS1 function. MicroRNA-155 (miR155) as a pro-inflammatory regulator could enhance macrophage inflammatory responses by targeting and degrading SOCS-1 [81, 82]. RNA virus infection induces miR-155 expression in macrophages via TLR/MyD88-independent and the inducible miR-155 feedback positively regulates host antiviral innate immune response by promoting type I IFN signaling via targeting SOCS1 [83, 84]. In human macrophages, miR-155 directly targets IL13Rα1 and reduces the levels of IL13Rα1 protein, leading to diminished activation of STAT6. MiR-155 affects the IL-13-dependent regulation of several genes (SOCS1, DC-SIGN, CCL18, CD23, and SERPINE) involved in the establishment of an M2/pro-Th2 phenotype in macrophages [85]. Further studies showed that miR-155 might promote the inflammation in atherosclerosis (AS) via the SOCS1-STAT3-PDCD4 axis, which increased the IL-6 and TNF-α expression in macrophages [86]. Thus we could find that miR-155-mediated SOCS1 downregulation is extremely associated with M1 polarization. As expected, Ma et al suggested that resveratrol exerts anti-inflammatory effects due to the upregulation of SOCS1 in macrophages, which is a potential target of miR-155 [82]. In contrast, inhibiting the STAT1-miR-155-SOCS1 signaling axis might enhance the development of tumor-promoting M2 macrophages in colon cancer [87]. These findings thus reveal a novel role of miR-155-SOCS1 pathway in the balance between pro-inflammatory M1 macrophages and anti-inflammatory M2 macrophages during different kinds of diseases [86]. Additionally, upregulation of miR-142-5p and downregulation of miR-130a-3p in macrophages play a pivotal role in the profibrogenic M2 effect of tissue fibrogenesis partially through targeting the SOCS1-STAT6 signaling [88]. SOCS1 is a key regulator of M1/M2 functions that coordinates with various cellular signaling pathways underlies the pathogenesis of many diseases, which could be developed as a useful therapy target (Figure 4).

### SOCS3

SOCS3 protein is commonly known to serve as a negative regulator of STAT3, which is the key physiological regulator in immune homeostasis and diseases pathogenesis [89–91]. SOCS3 has been shown to be induced by a wide variety of the cytokines and growth factors, such as IL-1β, 2, 4 and M-CSF [92, 93]. Unlike its closest homologue, SOCS-3 molecules differ greatly in their mechanism of function of SOCS-1. SOCS1 can inhibit activation of JAK by directly binding to JAK, whereas SOCS-3 sufficiently inhibits the action of JAK only in the presence of receptors, such as gp130 [94]. SOCS3 is a repressor of cytokine signaling which could inhibit the inflammatory genes expression in macrophages [95–97]. Recently, Sun et al investigated the anti-inflammatory effect of L. paracasei on the inhibition of TNF-α, IL-1β and IL-6 expressions by PBMC and THP-1 cell line owing to the expression of negative regulators of the NF-κB signaling pathway, including the SOCS1, SOCS3 [97]. Recombinant human IL-37 (rhIL-37) has both preventive and therapeutic effects in gouty arthritis by enhancing the activation of SOCS3 to trigger multiple intracellular switches to block inflammation [98]. Of note, several reports are available for the roles of SOCS3 in modulating macrophages M1/M2 polarization governed by the transcriptional and post-transcriptional mechanisms [99–101]. For example, SOCS3-deficient macrophages exhibit heightened STAT3 activation and are polarized toward the M1 phenotype [102]. Intriguingly, M2 macrophage (expressing upregulated Arg-1 and SOCS3) could be switched from M1 through the apolipoprotein E (APOE) signaling via very-low-density lipoprotein receptor (VLDL-R) or APOE receptor-2 (APOER2) [103]. These results collectively imply that SOCS3 is involved in repressing the M1 pro-inflammatory phenotype, thereby ameliorating inflammatory responses in macrophages [104]. SOCS3 is highly and preferentially expressed in hapten-induced contact hypersensitivity (CHS), which promotes the M2 polarization and participates the attenuation of CHS by suppressing MMP12 production [105]. Reprogramming macrophages to M2c subtype effectively suppressed the inflammation and fibroproliferarion in acute lung injury (ALI) partially mediated by activating the JAK1/STAT3/SOCS3 signaling pathway due to the production of IL-10 [106]. Collectively, we concluded that STAT3 and SOCS3 signaling are extremely required for the pro- and anti-inflammatory effects of macrophages, respectively.

There is also increasing evidence suggests that SOCS3 is involved in suppressing the M2 polarization and reciprocally promoting the M1 polarization. For example, macrophages lacking the SOCS3 gene or carrying a mutation of the SOCS3-binding site in gp130, the production of TNF and IL-12 is suppressed by the anti-inflammatory response originated from IL-6 signaling [107]. The *car*bohydrate *k*inase-*like* protein (CARKL) is rapidly downregulated *in vitro* and *in vivo* upon LPS stimulation in both mice and humans and the CARKL-dependent metabolic reprogramming is required for proper M2 polarization, which is associated with enhanced STAT3 phosphorylation and decreased SOCS3 expression, without influencing SOCS1 levels [108]. Rat bone marrow-derived macrophages (BMDM) in incubation with IFN-γ and LPS suppressed SOCS1 while uniquely polarizing macrophages to SOCS3 expressing macrophages, which proposing that SOCS3 is essential for development of M1 macrophages. In contrast, knockdown of SOCS3 by siRNA had enhanced STAT3 activity; induction of macrophage mannose receptor, Arg-1 and SOCS1 [109]. Myeloid-restricted SOCS3 deletion (*Socs3*^Lyz2cre^) resulted in resistance to LPS-induced endotoxic shock and observed striking bias toward M2 macrophages in *Socs3*^Lyz2cre^ mice, enhanced regulatory T (Treg) cell recruitment by *Socs3*^Lyz2cre^ cells coincided with enhanced interleukin-4 (IL-4) plus IL-13-induced STAT6 phosphorylation in *Socs3*^Lyz2cre^ macrophages. [110] Taken together, these observations strongly suggest that phenotypic and functional heterogeneity of SOCS3-expressing macrophages within different diseases might be dependent on the specific local microenvironments. Finally, understanding how the M1/M2 polarization occurs *in vivo*, which is essential for designing pharmacologic and genetic approaches that employed to treat various diseases (Figure 4).

### Other SOCS proteins

The function as well as the mechanism of SOCS-2 has been reported to be involved in the SHP2-binding site of activated growth hormone (GH) receptors, and it attenuates GH signaling by inhibiting the activation of JAK2 and STAT5b axis [111]. SOCS2 is considered as essential controller of macrophage activation and function. Genetic studies using transgenic approaches have shown that SOCS2 over-expressing transgenic (SOCS2Tg) mice showed functional improvement of anti-inflammatory response with the increased numbers of CD11b^+^CD206^+^ M2 macrophages than wildtype littermates following mild or moderately severe traumatic brain injury (TBI) [112]. As expected, M1 population was enriched in SOCS2(−/−) mice and the altered polarization coincided with enhanced IFN-γ-induced STAT1 activation in SOCS2(−/−) macrophages [110]. Therefore, SCOS2 is known as the M2 marker and the mRNA levels are downregulated with increasing disease severity in isolated lesions of atherosclerotic disease [113].

Previous studies have supported that miRNAs are functionally involved in macrophage polarization and function by the translational repression mechanism [114]. Up-regulation of let-7b is characteristic of prostatic TAMs (Tumor-associated macrophages), which targeting of the SOCS4 3′ untranslated region and inhibition of SOCS4 promoted phosphorylation of STAT3 and STAT6 [115]. Signal activation of Jak2/STAT3/STAT6 pathway could exert an impact on the M2 polarization [116]. Given the aforementioned observations, the detailed analysis of the mechanisms of SOCS4 underlying macrophages polarization merits consideration. In addition, SOCS5 was particularly suppressed in the M1-IFNγ status confirmed by the RNA-Seq Data with the genome-wide analysis of the expression of transcription factors (TFs) in SOCS families [117]. Of note, SOCS5 recently has been shown to contribute to M1 polarization by binding the IL-4Rα and blocking STAT6 phosphorylation [118, 119]. The role of SOCS6 and SOCS7 in M1/M2 polarization is still obscure. For example, miR-199a-3p accumulation was associated with the P53 induction in renal fibrosis and SOCS7 was identified as a target gene of miR-199a-3p. Silencing of SOCS7 promoted STAT3 activation and the infiltration of macrophage was suppressed in p53-KO mice [120]. These lesser-studied SOCS, such as SOCS6 and SOCS7 may play a yet undefined role in macrophage polarization and is needed to be further investigated [121].

## CONCLUDING REMARKS

Altogether, M1/M2 subtypes might appear to be indispensable for fine-tuning the host responses to the various pathogens in different diseases. An understanding of how to program the macrophage polarization process is a keystone of deciphering homeostasis and disease pathogenesis. heThese observations that SOCS proteins participate in directing the dynamics of macrophage polarization have drawn attention to SOCS-dependent macrophage functions as a potential therapeutic target. Collectively, more in-depth analysis of how SOCS members operate the M1/M2 polarization is vital for developing novel strategies in the antiviral, antibacterial and antitumor responses.

## Abbreviations

SOCS: suppressors of cytokine signaling
JAK: Janus kinases
ILs: interleukins
STAT: signal transducers and activators of transcription
IFNs: interferons
CIS: Src homology 2 (SH2)-containing protein
TAMs: tumor associated macrophages
RBX2: RING-box-2
KIR: kinase inhibitory region
TAMs: tumor associated macrophages
MAO-A: monoamine oxidase A
LPS: lipopolysaccharide
GM-CSF: granulocyte macrophage colony-stimulating factor
DNMT1: DNA methyltransferases1
TFs: transcription factors
M-CSF: macrophage colony-stimulating factor
PHD3: prolyl hydroxylase
AMs: alveolar macrophages
PDGF: platelet-derived growth factor
MOG: myelin oligodendrocyte glycoprotein
EAE: experimental autoimmune encephalomyelitis
APOE: apolipoprotein E
VLDL-R: very-low-density lipoprotein receptor
CARKL: carbohydrate kinase-like protein
GH: growth hormone
iNOS: inducible nitric oxide synthase
Arg-1: arginine-1.
